# 3-Acetyl-1-(4-methyl­phen­yl)thio­urea

**DOI:** 10.1107/S1600536812026244

**Published:** 2012-06-16

**Authors:** B. Thimme Gowda, Sabine Foro, Sharatha Kumar

**Affiliations:** a Department of Chemistry, Mangalore University, Mangalagangotri 574 199, Mangalore, India; b Institute of Materials Science, Darmstadt University of Technology, Petersenstrasse 23, D-64287 Darmstadt, Germany

## Abstract

The asymmetric unit of the title compound, C_10_H_12_N_2_OS, contains two independent mol­ecules. In both mol­ecules, the conformations of the two N—H bonds are *anti* to each other. Furthermore, the conformations of the amide C=S bonds and the C=O bonds are *anti* to each other. The dihedral angles between the benzene ring and the side chain are 52.8 (1) and 68.0 (1)° in the two independent mol­ecules. An intra­molecular N—H⋯O hydrogen bond occurs in both independent mol­ecules. In the crystal, mol­ecules are linked into infinite chains along the *a* axis through a series of N—H⋯O and N—H⋯S hydrogen bonds.

## Related literature

For studies on the effects of substituents on the structures and other aspects of *N*-(ar­yl)-amides, see: Gowda & Weiss (1994 ▶); Shahwar *et al.* (2012 ▶), of *N*-(ar­yl)-methane­sulfonamides, see: Gowda *et al.* (2007 ▶), of *N*-(ar­yl)-aryl­sulfonamides, see: Gowda *et al.* (2005 ▶) and of *N*-chloro­aryl­sulfonamides, see: Jyothi & Gowda (2004 ▶); Shetty & Gowda (2004 ▶).
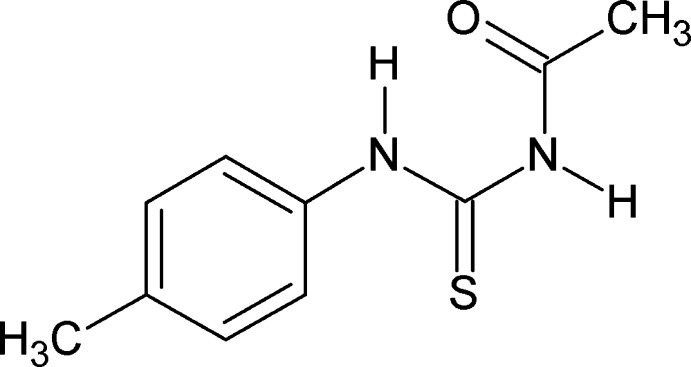


## Experimental

### 

#### Crystal data

C_10_H_12_N_2_OS
*M* *_r_* = 208.28
Triclinic, 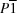

*a* = 9.1623 (8) Å
*b* = 10.130 (1) Å
*c* = 13.446 (1) Å
α = 73.212 (9)°
β = 70.276 (8)°
γ = 66.772 (8)°
*V* = 1061.90 (16) Å^3^
*Z* = 4
Mo *K*α radiation
μ = 0.27 mm^−1^
*T* = 293 K
0.36 × 0.32 × 0.24 mm

#### Data collection

Oxford Diffraction Xcalibur diffractometer with a Sapphire CCD detector
Absorption correction: multi-scan (*CrysAlis RED*; Oxford Diffraction, 2009 ▶) *T*_min_ = 0.908, *T*_max_ = 0.937
7674 measured reflections
4293 independent reflections
3267 reflections with *I* > 2σ(*I*)
*R*_int_ = 0.013

#### Refinement

*R*[*F* ^2^ > 2σ(*F* ^2^)] = 0.041
*wR*(*F* ^2^) = 0.112
*S* = 1.00
4293 reflections
257 parameters
H atoms treated by a mixture of independent and constrained refinement
Δρ_max_ = 0.17 e Å^−3^
Δρ_min_ = −0.26 e Å^−3^

### 

Data collection: *CrysAlis CCD* (Oxford Diffraction, 2009 ▶); cell refinement: *CrysAlis CCD*; data reduction: *CrysAlis RED* (Oxford Diffraction, 2009 ▶); program(s) used to solve structure: *SHELXS97* (Sheldrick, 2008 ▶); program(s) used to refine structure: *SHELXL97* (Sheldrick, 2008 ▶); molecular graphics: *PLATON* (Spek, 2009 ▶); software used to prepare material for publication: *SHELXL97*.

## Supplementary Material

Crystal structure: contains datablock(s) I, global. DOI: 10.1107/S1600536812026244/nc2284sup1.cif

Structure factors: contains datablock(s) I. DOI: 10.1107/S1600536812026244/nc2284Isup2.hkl

Supplementary material file. DOI: 10.1107/S1600536812026244/nc2284Isup3.cml

Additional supplementary materials:  crystallographic information; 3D view; checkCIF report

## Figures and Tables

**Table 1:** Hydrogen-bond geometry (Å, °)

*D*—H⋯*A*	*D*—H	H⋯*A*	*D*⋯*A*	*D*—H⋯*A*
N1—H1*N*⋯O1	0.84	2.02	2.674 (2)	134
N1—H1*N*⋯O2^i^	0.84	2.47	3.198 (2)	145
N2—H2*N*⋯S2^ii^	0.84	2.68	3.5058 (17)	169
N3—H3*N*⋯O2	0.86	1.97	2.661 (2)	137
N3—H3*N*⋯O1^i^	0.86	2.42	3.131 (2)	140
N4—H4*N*⋯S1^ii^	0.85	2.57	3.4078 (17)	169

Symmetry codes: (i) 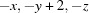; (ii) 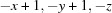.

**Fig. 1.**
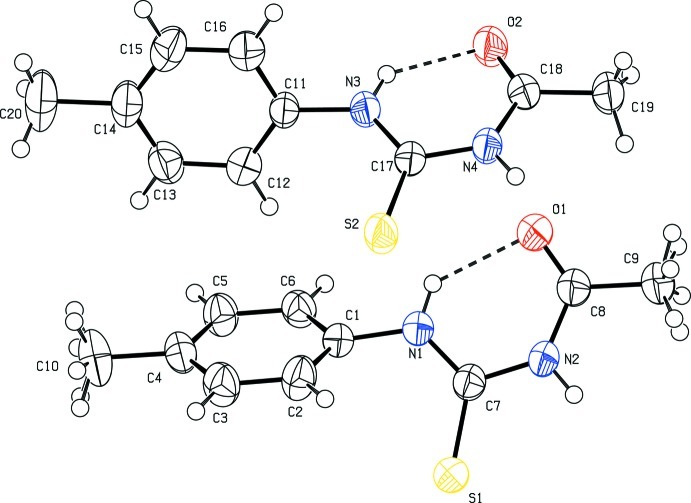
Molecular structure of the title compound, showing the atom labelling scheme and with displacement ellipsoids drawn at the 50% probability level. Please note that the H atoms in two methyl groups are disordered.

**Fig. 2.**
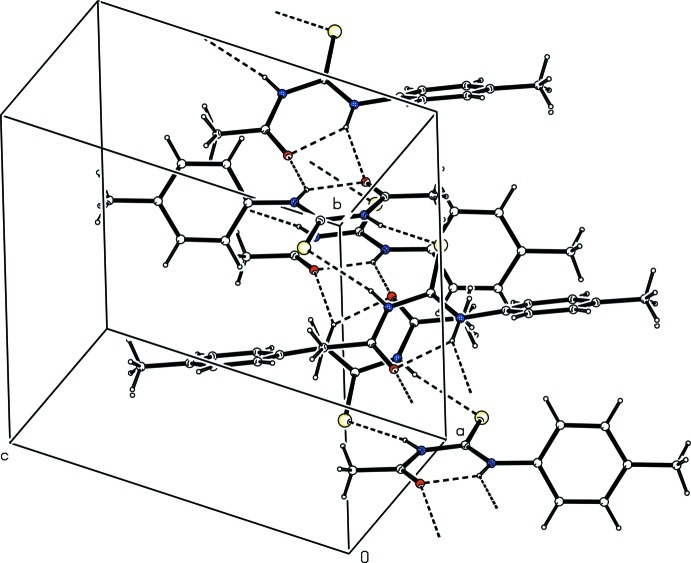
Molecular packing of the title compound with hydrogen bonding shown as dashed lines.

## supplementary crystallographic information

### Comment

Thiourea and its derivatives are widely used as precursors or intermediates towards the syntheisis of a variety of heterocyclic compounds. They are known to exhibit a wide variety of biological activities. As part of our studies on the substituent effects on the structures and other aspects of *N*-(aryl)-amides (Gowda & Weiss, 1994); *N*-(aryl)-methanesulfonamides (Gowda *et al.*, 2007); *N*-(aryl)-arylsulfonamides (Gowda *et al.*, 2005) and *N*-chloroarylsulfonamides (Jyothi & Gowda, 2004; Shetty & Gowda, 2004). in the present work, the crystal structure of 3-acetyl-1-(4-methylphenyl)thiourea has been determined (Fig. 1).

The asymmetric unit of the structure contains two independent molecules. The conformations of the two N—H bonds in the side chain are *anti* to each other and one of them is *anti* to the C═S in the urea segments and the other is *syn*, in both the molecules, similar to the *anti* conformation observed in 3-acetyl-1-(2-methylphenyl)thiourea (Shahwar *et al.*, 2012) Further, the conformations of the amide C═S and the C═O are *anti* to each other.

The side chains are oriented themselves with respect to the phenyl rings with the torsion angles of C2—C1—N1—C7 = 53.32 (32)° and C6—C1—N1—C7 = - 131.28 (24)° in molecule 1, and C12—C11—N3—C17 = - 67.14 (31)° and C16—C11—N3—C17 = 116.61 (26)° in molecule 2. The dihedral angles between the phenyl rings and the side chains are 52.8 (1)° and 68.0 (1)°, in the two independent molecules.

The amide oxygen and one of the NH hydrogen atoms exhibit both intra- and inter-molecular bifurcated hydrogen bonding. In the structure, series of N—H···O and N—H···S intermolecular hydrogen bonds pack the molecules into infinite chains (Table 1, Fig.2).

### Experimental

3-Acetyl-1-(4-methylphenyl)thiourea was synthesized by adding a solution of acetyl chloride (0.10 mol) in acetone (30 ml) dropwise to a suspension of ammonium thiocyanate (0.10 mol) in acetone (30 ml). The reaction mixture was refluxed for 30 min. After cooling to room temperature, a solution of 4-methylaniline (0.10 mol) in acetone (10 ml) was added and refluxed for 3 h. The reaction mixture was poured into acidified cold water. The precipitated title compound was recrystallized to constant melting point from acetonitrile. The purity of the compound was checked and characterized by its infrared spectrum.

Prism like yellow single crystals used in X-ray diffraction studies were grown in acetonitrile solution by slow evaporation of the solvent at room temperature.

### Refinement

H atoms bonded to C were positioned with idealized geometry using a riding model with the aromatic C—H = 0.93 Å and methyl C—H = 0.96 Å. The amino H atoms were freely refined with the N—H distances restrained to 0.86 (2) Å. All H atoms were refined with isotropic displacement parameters set at 1.2 *U*_eq_(C-aromatic, N) and 1.5 *U*_eq_(*C*-methyl) of the parent atom. In one of the two crystallographically independent molecules the H atoms of both methyl groups are disordered and were refined using a split model.

### Crystal data

**Table:**

C_10_H_12_N_2_OS	*Z* = 4
*M**_r_* = 208.28	*F*(000) = 440
Triclinic, *P*1	*D*_x_ = 1.303 Mg m^−^^3^
Hall symbol: -P 1	Mo *K*α radiation, λ = 0.71073 Å
*a* = 9.1623 (8) Å	Cell parameters from 3355 reflections
*b* = 10.130 (1) Å	θ = 2.6–27.8°
*c* = 13.446 (1) Å	µ = 0.27 mm^−^^1^
α = 73.212 (9)°	*T* = 293 K
β = 70.276 (8)°	Prism, yellow
γ = 66.772 (8)°	0.36 × 0.32 × 0.24 mm
*V* = 1061.90 (16) Å^3^	

### Data collection

**Table:**

Oxford Diffraction Xcalibur diffractometer with a Sapphire CCD detector	4293 independent reflections
Radiation source: fine-focus sealed tube	3267 reflections with *I* > 2σ(*I*)
Graphite monochromator	*R*_int_ = 0.013
Rotation method data acquisition using ω and phi scans	θ_max_ = 26.4°, θ_min_ = 2.6°
Absorption correction: multi-scan (*CrysAlis RED*; Oxford Diffraction, 2009)	*h* = −11→11
*T*_min_ = 0.908, *T*_max_ = 0.937	*k* = −11→12
7674 measured reflections	*l* = −9→16

### Refinement

**Table:**

Refinement on *F*^2^	Primary atom site location: structure-invariant direct methods
Least-squares matrix: full	Secondary atom site location: difference Fourier map
*R*[*F*^2^ > 2σ(*F*^2^)] = 0.041	Hydrogen site location: inferred from neighbouring sites
*wR*(*F*^2^) = 0.112	H atoms treated by a mixture of independent and constrained refinement
*S* = 1.00	*w* = 1/[σ^2^(*F*_o_^2^) + (0.0512*P*)^2^ + 0.3765*P*] where *P* = (*F*_o_^2^ + 2*F*_c_^2^)/3
4293 reflections	(Δ/σ)_max_ = 0.001
257 parameters	Δρ_max_ = 0.17 e Å^−^^3^
0 restraints	Δρ_min_ = −0.26 e Å^−^^3^

### Special details

**Table:**

Experimental. Absorption correction: CrysAlis RED (Oxford Diffraction, 2009) Empirical absorption correction using spherical harmonics, implemented in SCALE3 ABSPACK scaling algorithm.
Geometry. All e.s.d.'s (except the e.s.d. in the dihedral angle between two l.s. planes) are estimated using the full covariance matrix. The cell e.s.d.'s are taken into account individually in the estimation of e.s.d.'s in distances, angles and torsion angles; correlations between e.s.d.'s in cell parameters are only used when they are defined by crystal symmetry. An approximate (isotropic) treatment of cell e.s.d.'s is used for estimating e.s.d.'s involving l.s. planes.
Refinement. Refinement of *F*^2^ against ALL reflections. The weighted *R*-factor *wR* and goodness of fit *S* are based on *F*^2^, conventional *R*-factors *R* are based on *F*, with *F* set to zero for negative *F*^2^. The threshold expression of *F*^2^ > σ(*F*^2^) is used only for calculating *R*-factors(gt) *etc*. and is not relevant to the choice of reflections for refinement. *R*-factors based on *F*^2^ are statistically about twice as large as those based on *F*, and *R*- factors based on ALL data will be even larger.

### Fractional atomic coordinates and isotropic or equivalent isotropic displacement parameters (Å^2^)

**Table:**

	*x*	*y*	*z*	*U*_iso_*/*U*_eq_	Occ. (<1)
S1	0.28304 (7)	0.27948 (6)	0.08545 (5)	0.0639 (2)	
O1	0.19291 (19)	0.74868 (16)	−0.09138 (12)	0.0662 (5)	
N1	0.11169 (19)	0.56173 (17)	0.08639 (12)	0.0447 (4)	
H1N	0.0910	0.6488	0.0539	0.054*	
N2	0.32618 (19)	0.50464 (17)	−0.06329 (12)	0.0462 (4)	
H2N	0.4049	0.4361	−0.0901	0.055*	
C1	0.0100 (2)	0.5382 (2)	0.19255 (14)	0.0390 (4)	
C2	0.0770 (3)	0.4649 (2)	0.27913 (15)	0.0523 (5)	
H2	0.1903	0.4247	0.2685	0.063*	
C3	−0.0244 (3)	0.4514 (3)	0.38129 (16)	0.0549 (5)	
H3	0.0221	0.3993	0.4387	0.066*	
C4	−0.1927 (3)	0.5128 (2)	0.40096 (15)	0.0469 (5)	
C5	−0.2577 (2)	0.5863 (2)	0.31314 (15)	0.0494 (5)	
H5	−0.3709	0.6284	0.3238	0.059*	
C6	−0.1575 (2)	0.5985 (2)	0.20985 (15)	0.0439 (5)	
H6	−0.2038	0.6476	0.1520	0.053*	
C7	0.2348 (2)	0.4588 (2)	0.03716 (15)	0.0427 (4)	
C8	0.3017 (2)	0.6427 (2)	−0.12374 (15)	0.0467 (5)	
C9	0.4200 (3)	0.6519 (3)	−0.23205 (17)	0.0615 (6)	
H9A	0.4651	0.5585	−0.2539	0.092*	0.50
H9B	0.5070	0.6792	−0.2286	0.092*	0.50
H9C	0.3637	0.7237	−0.2831	0.092*	0.50
H9D	0.4254	0.7491	−0.2565	0.092*	0.50
H9E	0.3835	0.6284	−0.2819	0.092*	0.50
H9F	0.5268	0.5839	−0.2273	0.092*	0.50
C10	−0.3005 (3)	0.5015 (3)	0.51417 (17)	0.0720 (7)	
H10A	−0.2341	0.4686	0.5642	0.108*	0.50
H10B	−0.3557	0.4331	0.5255	0.108*	0.50
H10C	−0.3803	0.5955	0.5248	0.108*	0.50
H10D	−0.4127	0.5296	0.5121	0.108*	0.50
H10E	−0.2910	0.5650	0.5508	0.108*	0.50
H10F	−0.2664	0.4026	0.5515	0.108*	0.50
S2	0.33996 (7)	0.74388 (6)	0.20962 (4)	0.05795 (19)	
O2	0.12185 (17)	1.10683 (16)	−0.04421 (11)	0.0580 (4)	
N3	0.09598 (19)	0.98380 (17)	0.16188 (12)	0.0455 (4)	
H3N	0.0571	1.0519	0.1132	0.055*	
N4	0.32028 (19)	0.91449 (17)	0.02163 (11)	0.0450 (4)	
H4N	0.4169	0.8559	0.0027	0.054*	
C11	−0.0049 (2)	0.9812 (2)	0.26949 (14)	0.0403 (4)	
C12	−0.0728 (3)	0.8725 (2)	0.32034 (16)	0.0505 (5)	
H12	−0.0475	0.7950	0.2868	0.061*	
C13	−0.1786 (3)	0.8790 (2)	0.42149 (16)	0.0545 (5)	
H13	−0.2251	0.8056	0.4550	0.065*	
C14	−0.2173 (3)	0.9914 (3)	0.47410 (16)	0.0552 (6)	
C15	−0.1460 (3)	1.0980 (3)	0.42215 (18)	0.0644 (6)	
H15	−0.1689	1.1741	0.4565	0.077*	
C16	−0.0411 (3)	1.0949 (2)	0.32005 (17)	0.0555 (5)	
H16	0.0045	1.1688	0.2860	0.067*	
C17	0.2422 (2)	0.8888 (2)	0.12968 (14)	0.0407 (4)	
C18	0.2596 (2)	1.0195 (2)	−0.05967 (15)	0.0449 (5)	
C19	0.3800 (3)	1.0180 (3)	−0.16731 (16)	0.0588 (6)	
H19A	0.3222	1.0535	−0.2225	0.088*	
H19B	0.4529	0.9200	−0.1724	0.088*	
H19C	0.4424	1.0796	−0.1762	0.088*	
C20	−0.3339 (3)	0.9966 (4)	0.58516 (19)	0.0884 (9)	
H20A	−0.3021	0.9033	0.6307	0.133*	
H20B	−0.4437	1.0194	0.5802	0.133*	
H20C	−0.3299	1.0702	0.6149	0.133*	

### Atomic displacement parameters (Å^2^)

**Table:**

	*U*^11^	*U*^22^	*U*^33^	*U*^12^	*U*^13^	*U*^23^
S1	0.0638 (4)	0.0405 (3)	0.0528 (3)	−0.0047 (3)	0.0089 (3)	−0.0042 (2)
O1	0.0649 (10)	0.0448 (8)	0.0526 (9)	−0.0031 (7)	0.0073 (7)	−0.0039 (7)
N1	0.0463 (9)	0.0382 (8)	0.0347 (8)	−0.0074 (7)	−0.0010 (7)	−0.0051 (7)
N2	0.0438 (9)	0.0418 (9)	0.0361 (8)	−0.0060 (7)	0.0016 (7)	−0.0082 (7)
C1	0.0430 (10)	0.0373 (10)	0.0319 (9)	−0.0114 (8)	−0.0050 (8)	−0.0071 (7)
C2	0.0394 (10)	0.0669 (14)	0.0403 (11)	−0.0061 (10)	−0.0091 (9)	−0.0122 (10)
C3	0.0573 (13)	0.0650 (14)	0.0363 (10)	−0.0137 (11)	−0.0170 (10)	−0.0035 (10)
C4	0.0523 (12)	0.0563 (12)	0.0333 (10)	−0.0267 (10)	−0.0036 (9)	−0.0060 (9)
C5	0.0384 (10)	0.0613 (13)	0.0437 (11)	−0.0177 (9)	−0.0055 (9)	−0.0068 (9)
C6	0.0441 (11)	0.0460 (11)	0.0360 (10)	−0.0115 (9)	−0.0116 (8)	−0.0023 (8)
C7	0.0392 (10)	0.0435 (10)	0.0374 (10)	−0.0090 (8)	−0.0051 (8)	−0.0079 (8)
C8	0.0440 (11)	0.0457 (11)	0.0407 (10)	−0.0116 (9)	−0.0036 (8)	−0.0068 (9)
C9	0.0586 (13)	0.0566 (13)	0.0470 (12)	−0.0169 (11)	0.0074 (10)	−0.0046 (10)
C10	0.0716 (16)	0.106 (2)	0.0387 (12)	−0.0479 (16)	0.0015 (11)	−0.0064 (12)
S2	0.0506 (3)	0.0539 (3)	0.0376 (3)	0.0033 (2)	−0.0053 (2)	0.0017 (2)
O2	0.0486 (8)	0.0539 (9)	0.0413 (8)	0.0010 (7)	−0.0047 (6)	0.0015 (6)
N3	0.0431 (9)	0.0417 (9)	0.0312 (8)	−0.0027 (7)	−0.0026 (7)	−0.0012 (7)
N4	0.0415 (9)	0.0421 (9)	0.0315 (8)	−0.0021 (7)	−0.0012 (7)	−0.0043 (7)
C11	0.0366 (9)	0.0425 (10)	0.0300 (9)	−0.0059 (8)	−0.0034 (7)	−0.0052 (8)
C12	0.0517 (12)	0.0477 (11)	0.0447 (11)	−0.0141 (10)	−0.0032 (9)	−0.0117 (9)
C13	0.0503 (12)	0.0586 (13)	0.0459 (12)	−0.0225 (11)	−0.0029 (9)	−0.0016 (10)
C14	0.0462 (12)	0.0713 (15)	0.0348 (10)	−0.0125 (11)	−0.0013 (9)	−0.0106 (10)
C15	0.0724 (16)	0.0678 (15)	0.0493 (13)	−0.0217 (13)	0.0020 (11)	−0.0276 (11)
C16	0.0617 (13)	0.0519 (12)	0.0479 (12)	−0.0228 (11)	−0.0007 (10)	−0.0117 (10)
C17	0.0423 (10)	0.0390 (10)	0.0325 (9)	−0.0091 (8)	−0.0045 (8)	−0.0065 (8)
C18	0.0475 (11)	0.0417 (10)	0.0350 (10)	−0.0113 (9)	−0.0051 (8)	−0.0031 (8)
C19	0.0569 (13)	0.0578 (13)	0.0353 (10)	−0.0061 (10)	−0.0005 (9)	−0.0017 (9)
C20	0.0750 (18)	0.125 (3)	0.0440 (13)	−0.0295 (18)	0.0122 (12)	−0.0219 (15)

### Geometric parameters (Å, º)

**Table:**

S1—C7	1.672 (2)	C10—H10D	0.9600
O1—C8	1.212 (2)	C10—H10E	0.9600
N1—C7	1.329 (2)	C10—H10F	0.9600
N1—C1	1.429 (2)	S2—C17	1.6777 (19)
N1—H1N	0.8407	O2—C18	1.211 (2)
N2—C8	1.373 (2)	N3—C17	1.319 (2)
N2—C7	1.387 (2)	N3—C11	1.429 (2)
N2—H2N	0.8403	N3—H3N	0.8569
C1—C6	1.373 (3)	N4—C18	1.381 (2)
C1—C2	1.381 (3)	N4—C17	1.385 (2)
C2—C3	1.378 (3)	N4—H4N	0.8494
C2—H2	0.9300	C11—C12	1.373 (3)
C3—C4	1.377 (3)	C11—C16	1.377 (3)
C3—H3	0.9300	C12—C13	1.380 (3)
C4—C5	1.386 (3)	C12—H12	0.9300
C4—C10	1.510 (3)	C13—C14	1.378 (3)
C5—C6	1.384 (3)	C13—H13	0.9300
C5—H5	0.9300	C14—C15	1.376 (3)
C6—H6	0.9300	C14—C20	1.516 (3)
C8—C9	1.496 (3)	C15—C16	1.386 (3)
C9—H9A	0.9600	C15—H15	0.9300
C9—H9B	0.9600	C16—H16	0.9300
C9—H9C	0.9600	C18—C19	1.495 (3)
C9—H9D	0.9600	C19—H19A	0.9600
C9—H9E	0.9600	C19—H19B	0.9600
C9—H9F	0.9600	C19—H19C	0.9600
C10—H10A	0.9600	C20—H20A	0.9600
C10—H10B	0.9600	C20—H20B	0.9600
C10—H10C	0.9600	C20—H20C	0.9600
			
C7—N1—C1	125.49 (16)	C4—C10—H10D	109.5
C7—N1—H1N	118.6	H10A—C10—H10D	141.1
C1—N1—H1N	115.9	H10B—C10—H10D	56.3
C8—N2—C7	129.02 (16)	H10C—C10—H10D	56.3
C8—N2—H2N	117.5	C4—C10—H10E	109.5
C7—N2—H2N	113.4	H10A—C10—H10E	56.3
C6—C1—C2	119.26 (17)	H10B—C10—H10E	141.1
C6—C1—N1	119.26 (16)	H10C—C10—H10E	56.3
C2—C1—N1	121.32 (17)	H10D—C10—H10E	109.5
C3—C2—C1	119.90 (19)	C4—C10—H10F	109.5
C3—C2—H2	120.1	H10A—C10—H10F	56.3
C1—C2—H2	120.1	H10B—C10—H10F	56.3
C4—C3—C2	121.96 (19)	H10C—C10—H10F	141.1
C4—C3—H3	119.0	H10D—C10—H10F	109.5
C2—C3—H3	119.0	H10E—C10—H10F	109.5
C3—C4—C5	117.32 (17)	C17—N3—C11	126.15 (16)
C3—C4—C10	120.85 (19)	C17—N3—H3N	116.6
C5—C4—C10	121.83 (19)	C11—N3—H3N	117.2
C6—C5—C4	121.35 (18)	C18—N4—C17	128.49 (16)
C6—C5—H5	119.3	C18—N4—H4N	115.9
C4—C5—H5	119.3	C17—N4—H4N	115.5
C1—C6—C5	120.19 (18)	C12—C11—C16	119.91 (18)
C1—C6—H6	119.9	C12—C11—N3	121.24 (17)
C5—C6—H6	119.9	C16—C11—N3	118.75 (18)
N1—C7—N2	116.88 (17)	C11—C12—C13	119.65 (19)
N1—C7—S1	124.99 (15)	C11—C12—H12	120.2
N2—C7—S1	118.11 (14)	C13—C12—H12	120.2
O1—C8—N2	122.36 (17)	C14—C13—C12	121.8 (2)
O1—C8—C9	122.79 (19)	C14—C13—H13	119.1
N2—C8—C9	114.85 (17)	C12—C13—H13	119.1
C8—C9—H9A	109.5	C15—C14—C13	117.53 (19)
C8—C9—H9B	109.5	C15—C14—C20	121.5 (2)
H9A—C9—H9B	109.5	C13—C14—C20	121.0 (2)
C8—C9—H9C	109.5	C14—C15—C16	121.8 (2)
H9A—C9—H9C	109.5	C14—C15—H15	119.1
H9B—C9—H9C	109.5	C16—C15—H15	119.1
C8—C9—H9D	109.5	C11—C16—C15	119.4 (2)
H9A—C9—H9D	141.1	C11—C16—H16	120.3
H9B—C9—H9D	56.3	C15—C16—H16	120.3
H9C—C9—H9D	56.3	N3—C17—N4	116.44 (16)
C8—C9—H9E	109.5	N3—C17—S2	125.08 (14)
H9A—C9—H9E	56.3	N4—C17—S2	118.47 (13)
H9B—C9—H9E	141.1	O2—C18—N4	122.67 (17)
H9C—C9—H9E	56.3	O2—C18—C19	123.20 (18)
H9D—C9—H9E	109.5	N4—C18—C19	114.12 (17)
C8—C9—H9F	109.5	C18—C19—H19A	109.5
H9A—C9—H9F	56.3	C18—C19—H19B	109.5
H9B—C9—H9F	56.3	H19A—C19—H19B	109.5
H9C—C9—H9F	141.1	C18—C19—H19C	109.5
H9D—C9—H9F	109.5	H19A—C19—H19C	109.5
H9E—C9—H9F	109.5	H19B—C19—H19C	109.5
C4—C10—H10A	109.5	C14—C20—H20A	109.5
C4—C10—H10B	109.5	C14—C20—H20B	109.5
H10A—C10—H10B	109.5	H20A—C20—H20B	109.5
C4—C10—H10C	109.5	C14—C20—H20C	109.5
H10A—C10—H10C	109.5	H20A—C20—H20C	109.5
H10B—C10—H10C	109.5	H20B—C20—H20C	109.5

### Hydrogen-bond geometry (Å, º)

**Table:**

*D*—H···*A*	*D*—H	H···*A*	*D*···*A*	*D*—H···*A*
N1—H1*N*···O1	0.84	2.02	2.674 (2)	134
N1—H1*N*···O2^i^	0.84	2.47	3.198 (2)	145
N2—H2*N*···S2^ii^	0.84	2.68	3.5058 (17)	169
N3—H3*N*···O2	0.86	1.97	2.661 (2)	137
N3—H3*N*···O1^i^	0.86	2.42	3.131 (2)	140
N4—H4*N*···S1^ii^	0.85	2.57	3.4078 (17)	169

Symmetry codes: (i) −*x*, −*y*+2, −*z*; (ii) −*x*+1, −*y*+1, −*z*.
